# Application of the Mutant Libraries for *Candida albicans* Functional Genomics

**DOI:** 10.3390/ijms232012307

**Published:** 2022-10-14

**Authors:** Ruina Wang, Jiacun Liu, Yu Liu, Quanzhen Lv, Lan Yan

**Affiliations:** School of Pharmacy, Naval Medical University, 325 Guohe Road, Shanghai 200433, China

**Keywords:** *Candida albicans*, mutant library, functional genomics, phenotype, virulence, genetic screening

## Abstract

*Candida albicans* is a typical opportunistic pathogen in humans that causes serious health risks in clinical fungal infections. The construction of mutant libraries has made remarkable developments in the study of *C. albicans* molecular and cellular biology with the ongoing advancements of gene editing, which include the application of CRISPR-Cas9 and novel high-efficient transposon. Large-scale genetic screens and genome-wide functional analysis accelerated the investigation of new genetic regulatory mechanisms associated with the pathogenicity and resistance to environmental stress in *C. albicans*. More importantly, sensitivity screening based on *C. albicans* mutant libraries is critical for the target identification of novel antifungal compounds, which leads to the discovery of Sec7p, Tfp1p, Gwt1p, Gln4p, and Erg11p. This review summarizes the main types of *C. albicans* mutant libraries and interprets their applications in morphogenesis, biofilm formation, fungus–host interactions, antifungal drug resistance, and target identification.

## 1. Introduction

*Candida albicans* is one of the most prevalent fungal pathogens in human beings causing infectious diseases ranging from superficial mucosal and dermal infections to life-threatening systemic infections with a mortality rate of nearly 40% [1,2]. The typical biological feature of *C. albicans* is the diversity and plasticity of its morphology. The yeast-hyphal transition and white-opaque switching of *C. albicans* contribute to its pathogenicity and survival in host [3,4]. In addition to morphological transformation, *C. albicans* can also form biofilms, leading to resistance to antifungal drugs [5]. Therefore, it is critical to investigate the biological properties and identify the main genes in pathogenicity and drug resistance in order to discover novel antifungal strategies. Genome-wide genetic screening is a powerful high-throughput approach. Multiple types of *C. albicans* gene mutant libraries have been constructed, including gene knockout libraries [6,7,8,9], transposon insertion libraries [10,11,12,13,14,15,16,17,18,19], and the conditional expression libraries [20,21,22,23,24] (Table 1 lists the 21 mutant libraries included in this review). These libraries can be comprehensively used to characterize genes relevant to morphogenesis, biofilm formation, and fungus–host interaction, to analyze genetic regulatory mechanisms and to identify potential targets of the antifungal agents. This approach establishes a theoretical foundation for finding new ways to prevent and treat the fungal infections.

## 2. Methods Used in the Mutant Library Construction in *C. albicans*

### 2.1. Gene Disruption

**Figure 1:**
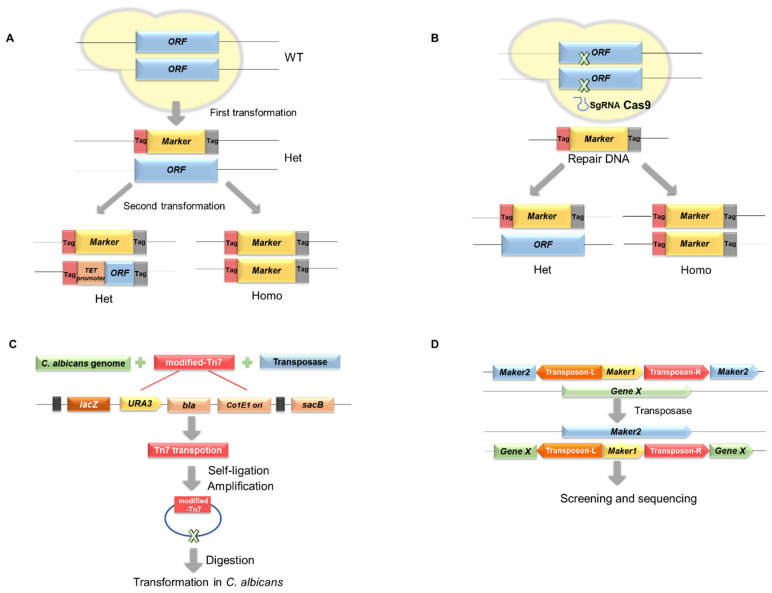
Methods used to construct the mutant library in *C. albicans.* (**A**,**B**) Schematic description of mutant library construction based on homologous recombination. (**A**) Traditional methods of homologous recombination used in gene deletion and conditional gene expression. (**B**) Schematic description of CRISPR/Cas9 technology. The commonly used markers include neutral auxotrophic markers (*URA3*, *HIS1*, *ARG4*, and *LEU2*) or drug-resistant marker (*SAT1*). Specific DNA barcodes are usually designed in the upstream and downstream of the markers, which could be used to distinguish each mutant by high-throughput sequencing. (**C**) Schematic description of modified-transposon mutagenesis [19]. *C. albicans* genomic DNA fragments digested with restriction enzyme were mixed with the Tn7 transposase and plasmid containing a modified Tn7 transposon. After the transposition, the modified Tn7 transposon inserted genomic DNA was ligated, and then transformed to *E. coli*. The plasmids were amplified and digested with restriction enzyme. Then the linear DNA fragments were transformed to *C. albicans* strain, which could integrate into the *C. albicans* genome by homologous recombination. The insertion mutants were collected and used as a heterozygous mutant library. (**D**) Transposition-mediated insertion mutant library construction in haploid *C. albicans*. Insertion of a transposon into marker2 results in mutation and loss of resistance. The transposition was initiated by the conditional expressed transposase. The transposed strains recovered the integrity of marker2, which could be used to screen positive clones. Het, heterozygous; Homo, homozygous.

Gene knockout is a commonly used strategy for constructing homozygous mutant libraries. In *C. albicans*, gene knockout cassettes typically carry the neutral auxotrophic markers or nourseothricin resistance genes, including *URA3*, *HIS1*, *LEU2*, *ARG4,* and *SAT1*. This requires two consecutive rounds of transformation to a double (or triple) auxotrophic parental strain, which takes at least 2 weeks to obtain a homozygous gene knockout mutant. Using the auxotrophic markers, Noble et al. constructed the heterozygous deletion mutant library (using *HIS1* and *LEU3* marker) [9], the homozygous deletion library (using *HIS1* and *LEU2* marker) [16], and the transcriptional regulator knockouts library (using *HIS1*, *LEU2,* and *ARG4* marker) [6] (Figure 1A). However, some auxotrophic markers, for example *URA3*, affect the growth and pathogenesis in *C. albicans*, leading to disturbance in investigation of certain genes’ biological function. Therefore, researchers started to use the nourseothricin resistance marker, *SAT1*. This marker has no significant effect on the growth or hyphae formation [28], which has been extensively applied in the gene editing in *C. albicans*. CRISPR/Cas9 is a recently utilized technique for gene knockout and mutation, which is efficient, time-saving, and recyclable. It only takes one transformation to obtain the heterozygous and homozygous mutant at the same time [29,30] (Figure 1B). Moreover, a CRISPR interference (CRISPRi) system is able to transcriptionally repress the target gene by a nuclease-dead Cas9 protein fusing to the target genetic promoter. This system can be applied to repress essential genes [31].

CRISPR-Cas9 can be used not only for the single gene editing but also for the construction of high-throughput mutant libraries. At present, large-scale and high-throughput genetic screens based on CRISPR-Cas9 have been achieved in mammalian cells through the transformation of sgRNA libraries which is commercially available [32,33]. In the studies of pathogenic fungi, Li et al., 2010, exploited a similar method called restriction/ligation coupled with Agrobacterium-mediated transformation (RELATe), which generated sgRNA libraries from *Cryptococcus neoformans* genomic DNA and covered 98% of the protein-coding genes [34]. In *C. albicans*, the genetic screen based on CRISPR-Cas9 and sgRNA libraries has not been reported, which indicated that the construction of sgRNA libraries, high-efficient transformation, recovery, and sequencing of sgRNA should be further explored.

### 2.2. Transposon Insertion

Transposon-mediated mutagenesis is a widely used genetic tool in bacteria and yeast [35]. Currently, several heterozygous [10,18,19] and homozygous insertion libraries [12,13] have been constructed in diploid *C. albicans* by using of bacterial Tn7 or Tn5 transposons. The Tn5 or Tn7 transposon combined with the screening marker *UAU1* or *URA3* can be transferred into the genomic DNA of *C. albicans* in vitro, and then, the mutagenized library is transferred into *E. coli* for amplification and finally transformed into *C. albicans*, where the transposons are integrated into the chromosome by homologous recombination with the flanking genomic DNA. This method is time-consuming and labor-intensive (Figure 1C). Gao J et al., 2018, devised a *PiggyBac* (PB)-based haploid *C. albicans* mutagenesis technique that is both easy and effective [14]. The PB transposon was originally obtained from the cabbage looper moth *Trichoplusiani* (*T.ni*). The PB transposon system contains a unique transposase that can recognize the inverted terminal repeats (ITRs) and can be accurately deleted from donor DNA and integrated into TTAA targets in genome [36]. The PB system simplifies not only insertion and precise excision but also phenotype-genotype screens [37] (Figure 1D). MielichK et al., 2018, recently inserted the maize activator/dissociation transposon (Ac/Ds) into haploid *C. albicans*. Transposition in vivo was performed by using a modified Ac transposase/Ds-NAT1 two-element system in a highly stable haploid strain [15]. The Ac/Ds transposon, unlike PB, has no clear insertion site preference in maize or other organisms, including *Saccharomyces cerevisiae*, and can quickly generate enormous libraries of random insertion mutants in a single strain background [38]. However, due to frequent auto-diploidization, the system currently has fewer applications [14]. In the construction of transposon insertion mutation library, there are some problems, such as uncontrolled spontaneous transposition, DNA polymerase preference, and insertion site preference. These problems could lead to the inaccurate quantification and uneven genome coverage of insertions.

### 2.3. Conditional Expression

Gene disruption and transposon insertion libraries mostly cover non-essential genes in *C. albicans*, whereas conditional expression libraries can be applied directly to functional research of essential genes [22]. The construction of conditional expression libraries requires suitable promoters. The most common promoter is a tetracycline promoter *TETp* (Figure 1A). Fu et al., 2008, created a library of 25 mutants by suppressing the genes encoding GPI-anchored surface proteins by the addition of doxycycline [39]. Meanwhile, Sahni et al., 2010, constructed 107 overexpression strains for transcription factors by *TET* inducible promoter [23]. Subsequently, the *TET* promoter was also used to construct the gene replacement and conditional expression libraries (GRACE) which contains 1152 strains by deleting one allele in *C. albicans* CaSS1 strain and then placing the other allele under the control of *TET* promoter [22]. Decades later, the GRACE library contains 2356 heterozygous deletion strains [21]. A derivative library from the GRACE collection contains 887 strains corresponding to nonessential genes, in which the tetracycline promoters have been looped out [27]. Alternatively, other promoters can be utilized to build conditional expression libraries. Using the *TET* promoter and the gluconeogenesis-induced *C. albicans PCK1* promoter, Chauvel et al., 2012, developed 384 overexpression strains [20], while Znaidi et al., 2018 later expanded the tetracycline-induced overexpression library to 572 mutant strains [25]. Flanagan et al., 2017, successfully constructed P*_ENO_TOR1*, P*_ENO_RHB1*, and P*_ENO_GTR1* strains by overexpressing these three genes in the TOR pathway in *C. albicans* under the control of enolase-encoding gene *ENO1* promoter [40]. The *ACT1* promoter and the galactose kinase *GAL1* promoter were used to create high-expression of *CDR1*, *CDR2*, and *MDR1* [41]. Using *GAL4* promoter and *SAT1* screening marker, Schillig, Morschhauser et al., 2013, developed a library of *C. albicans* strains conditional expressing all 82 zinc cluster transcription factors [24]. Conditional expression libraries allow researchers to compare phenotypes in both non-inhibitory and inhibitory conditions of genes, especially contributing to identify essential genes in *C. albicans*.

Overall, the *C. albicans* mutant library established by single gene editing and disruption mediated by transposon has its own advantages and disadvantages. The construction of *C. albicans* mutant library based on gene knockout or conditional expression one by one can provide reliable results for phenotype screening, and the mutant strains can be directly used for subsequent mechanistic studies, but the cost in primers, reagents, and time is huge. At the same time, knocking out or editing each gene is done once and for all, which can provide many conveniences for subsequent studies. In contrast, the construction of *C. albicans* mutant library based on transposon is mainly suitable for the heterozygous deletion of genes in diploid and the homozygous deletion of genes in haploid, which needs less time and costs. However, the reliability of differential genes obtained by high-throughput sequencing would be affected by the PCR amplification bias, and the accurate analysis of sequencing data, which always result in many false positive results. In addition, as for the obtained differential genes, it is still necessary to construct their knockout or conditional expression mutant to verify the relationship between the phenotypes and genes. Furthermore, when the *C. albicans* haploid is used to construct the mutant library, it is necessary to consider the defects of haploid in virulence and drug resistance, which would bring some limitations to its application [42].

## 3. Application of the *C. albicans* Mutant Library

### 3.1. Morphological Transformation

Yeast, pseudohyphae, and hyphal morphogenesis in *C. albicans* differs in gene expression profile during its adaptation to host environment changes and invasive process [43]. As a result, large-scale genetic screening combined with phenotypic analysis is an ideal tool to explore the important genes in morphological transformation in *C. albicans*.

Transposon-insertion libraries were first utilized to investigate biological function in *C. albicans*. The Tn7-*UAU1* strategy was used to create 217 homozygous insertion mutants, and *MDS3* was then discovered as a conserved regulator in pH-regulated hyphal formation [12]. Uhl et al., 2003, constructed over 18,000 strains of heterozygous deletion mutants by the Tn7-*URA3* transposon mutagenesis and finally identified 146 genes affecting switching between yeast and filamentous form by haploinsufficiency analysis. These genes encode proteins involved in nutrition sensing, signal transduction, transcriptional regulation, cytoskeletal architecture, and cell wall formation [19]. Furthermore, *VPS52* and *ARP2* were identified as essential genes in the yeast-to-hyphae transition by screening 4700 randomly inserted, homozygous mutants based on Tn-orf19.875-*UAU1* mutagenesis strategy. As one of the highly conserved *Arp2/3* complexes in *C. albicans* which nucleates actin filaments into branched networks, Arp2p is required for actin cytoskeleton, hyphal formation, and virulence in mice [13]. Bharucha N et al., 2011, created a double heterozygous mutant library containing 6528 independent strains based on the *cbk1*/*CBK1* heterozygous parental strain inserted by Tn7 transposition. The library was screened for filamentous defective strains on spider medium. A total of 41 transposon-derived mutations were discovered as potential synthetic genetic interactions with transcription factor Cbk1p. It was shown that regulation of *ACE2* and morphogenesis (RAM) and protein kinase A (PKA) pathways co-regulate a common set of genes during morphogenesis. The PKA regulated Efg1p is mainly localized in yeast phase cells while the RAM regulated Ace2p in the daughter nucleus of filamentous cells, demonstrating that Efg1p and Ace2p regulate a same collection of genes in distinct processes of morphogenesis. These approaches provide evidence for the application of transposon-based synthetic genetic interaction screens [10].

Homann et al., 2009 constructed a transcriptional regulator knockouts (TRKO) library by individually disrupting transcription factor and created a phenotype spectrum of 143 mutants by screening 55 growth conditions. Regulatory genes that control antifungal drug sensitivity, iron acquisition, and the ability to build complex multicellular colonies were identified [6]. Chauvel et al., 2012, searched an overexpressed gene library for regulators that alter morphogenesis and growth rate, which was enriched in protein kinases, protein phosphatases, transcription factors, and other signaling proteins [20]. The use of libraries based on transcription factors provides insight into the specific biological roles of certain regulators.

Using *HIS1* and *LEU2* markers, Noble et al., 2010, created about 3000 homozygous gene deletion strains impacting 674 genes. These mutants were used to genetic screen for virulence in mice, yeast-to-hyphae transformation, and cell proliferation rate. Contrary to expectations, among the 115 attenuated mutants, nearly half had normal colony morphology and growth rate, suggesting that these phenotypic traits are not strictly correlated with each other. In addition, further analysis of the mutants identified small molecule ceramide glucolipids and their metabolism-related genes are essential for the virulence in *C. albicans* [7].

The ability to switch between yeast and hyphal form is a major virulence feature in *C. albicans*. Hsp90 protein is one of the important regulators of morphogenesis, which can inhibit the filamentation at 30 °C in *C. albicans*. In order to identify genes important for filamentous growth in response to Hsp90p inhibitors, Hossain S et al., 2021, screened GRACE libraries [21,22] with the treatment of geldanamycin. A total of 54 strains were found to be deficient in mycelial growth under Hsp90 inhibition. The absence of mitochondrial gene *MSU1* (involved in the catabolism of mitochondrial RNA) and *SHY1* (involved in the biosynthesis of cytochrome c oxidase) led to resistance to Hsp90 inhibitors. They also found that *MSU1* and *SHY1* disruption could increase cell efflux and reduced sensitivity of *C. albicans* to fluconazole, terbinafine, and gepinacin. To further explore the efflux protein of Hsp90 inhibitors, an efflux pump gene deletion library was screened. It was shown that deletion of *YOR1* led to sensitivity to geldanamycin, while deletion of *CDR1* led to sensitivity to radicicol. Deletion of the transcription transporter gene *YOR1* and *CDR1* restored susceptibility to Hsp90p inhibitors and filamentous growth in homozygous deletion mutants [44]. The study applied gene library screening to explore the relationship between mitochondrial function and biomorphic switching, which can help researchers to better understand the complicated interaction between mitochondria, virulence phenotype, drug resistance, and cell signaling in fungal pathogens. In addition, white-to-opaque phenotypic transition is another morphological change in *C. albicans*, which plays role in adaption to environments. Yang SL et al., 2018, screened the PB transposon insertion library [14] and identified that the Sac7p and Rho1p are key elements of a novel signaling pathway required for the transition from white to opaque in *C. albicans* [45]. By using of *TET* promoter, a collection for overexpression of the 107 transcription factors was constructed and screened for the white-specific pheromone-induced transcription factor. Tec1p was discovered as the single transcription factor in the white cell pheromone response pathway in *C. albicans*. This new regulatory pathway consists of the upstream pheromone, receptor, trimeric G protein complex, and MAP kinase cascade. Tec1p is a transcription factor downstream of MAP kinase pathway, derived from the filamentous pathway; and white-specific downstream target genes of Tec1p are derived from biofilm biosynthesis pathway. Such pheromone response pathway’s evolution serves as a model for other similar pathways [23].

Understanding the regulatory pathways of morphogenesis will be helpful for the development of effective antifungal strategies, since the ability to regulate morphology is a major component of pathogenicity in *C. albicans*. The rapid development of functional genomic research tools provides vital clues to understand the distinctive regulatory mechanisms of morphogenesis in *C. albicans*. Progress in these areas will provide new potential targets for antifungal research.

### 3.2. Biofilm Formation

Biofilm is one of the important factors in microorganic pathogenicity and is clinically indispensable as the basis for infections related to implanted medical devices. Compared with planktonic cells, mature biofilms are more resistant to antifungal agents and host immune system [46], which poses great challenges for clinical prevention and treatment of candidiasis. With the development of genetic manipulation technology, the creation of genomic libraries has become increasingly essential in the field of biofilm research, particularly in understanding the signal regulation networks.

Transcriptional regulators-mediated genetic regulatory networks strictly regulate each stage of biofilm formation by responding to different environmental signals. Based on a parent strain DAY185, Nobile and Mitchell et al., 2005, screened a homozygous gene insertion library including 83 transcription factors. Bcr1p was found to activate cell surface protein and adhesin genes, which is dependent on its upstream regulation of mycelium regulator Tec1p. Since Bcr1p is required for biofilm formation but not hyphae, the results suggested that Bcr1p couples expression of cell-surface genes to hyphal formation [16]. By investigating the library composed of 165 transcription regulator deletion mutants, Bcr1p, Tec1p, Efg1p, Ndt80p, Rob1p, and Brg1p were further identified as master biofilm regulators. Deletion of any of these regulators resulted in deficient biofilm formation both in vitro and in rat infection models [47].

Fungal cell wall mediates pathogen’s interactions with host and environment. Adherence is the first step of biofilm formation. It was revealed that the adherence of yeast form cells to a substrate is governed by numerous regulatory signals by using the transcription factor library. Both regulator Bcr1p and Snf5p share several target genes. There are twelve transcription factors, including Try2p, Zap1p, Ada2p, etc., which regulate expression of more than one quarter of the cell surface targets of adherence regulators [48]. Norice et al., 2007, followed the Tn7-*UAU1* transposon insertion approach [12] to create 21 homozygous mutants with insertional mutagenesis of cell wall-related genes, demonstrating that Sun41p is essential for biofilm formation and caspofungin tolerance [17]. By using the same Tn7-*UAU1* transposon, the protein kinase mutant library was used to investigate the changes in sensitivity to several stresses. Eight protein kinases including Ckb1p, Ckb2p, Hsl1p, Hst7p, Prk1p, Tpk1p, Yck2p, and Yck3p showed different functions in cell wall biogenesis compared with *S. cerevisiae* [11].

The *Arp2/3* complex is revealed to be involved in fungal cell wall remodeling and biofilm formation through screening the GRACE library [22]. When Arp2/3 complex components were deleted, cell surface hydrophobicity was increased, and more cell wall chitin and β-glucans were exposed, followed by reduced biofilm formation in *C. albicans* [49]. The GRACE library [22] was further used to identify genes involved in biofilm formation under a strong biofilm-induced condition (RPMI-MOPS medium). A strain expressing very low level of the essential gene *ILS1*, encoding isoleucyl-tRNA synthetase, was incapable of yeast-phase growth but capable of producing the wild-type three-dimensional biofilm with decreased metabolic level [50]. Besides the classical regulatory clusters in biofilm, this study reveals a different regulation profile that depends on *ILS1*.

Shapiro RS et al., 2018, designed a CRISPR-Cas9-based gene drive platform to create a double-gene deletion library, targeting antifungal efflux and biofilm adhesion components, and revealed the complicated genetic relationship between drug resistance and biofilm formation in *C. albicans* [8]. Later, this platform was used to construct a set of single or double adhesion gene deletion mutants. In such mutant library were evaluated not only the ability of *C. albicans* in the *C. elegans* host but also the ability of morphological transformation and biofilm formation in vitro. They found that *ALS1*, *ALS3*, *ALS5*, *ALS7*, *ALS9*, *HYR1*, and *IFF4* null mutant are defective in hyphal growth and biofilm formation, but still virulent in *C. elegant*. Exploring the role of adhesive proteins in the virulence of *C. albicans* is of great significance for understanding fungal virulence and host interaction and provides more novel hypothesized targets for combined antifungal therapy [51]. Taken together, the application of gene mutant libraries facilitates the deep study of the genetic regulatory mechanisms of biofilm in *C. albicans*, laying a theoretical foundation for the clinical prevention and treatment of biofilm-triggered infection.

### 3.3. Fungus–Host Interaction

*C. albicans* is a commensal microorganism colonizing skin, oral mucosa, gastrointestinal mucosa, and vaginal mucosal in healthy individuals and an opportunistic pathogen in immunocompromised individuals [52]. The interaction between *C. albicans* and host immune cells determines the outcome of the infection. The genetic library has made great progress in understanding *Candida*–host interaction.

IL-1β, a proinflammatory cytokine, is essential for protecting the host from fungal infection. To explore the mechanism of filamentation-triggered IL-1β production, the TRKO library composed of 165 transcription factor deletion mutants [6] was screened in a mouse macrophage-like cell model. Mutants with nearly identical hyphal morphogenesis, such as *ndt80*Δ/Δ and *rob1*Δ/Δ mutants showing deficient hyphae, or *ahr1*Δ/Δ and *upc2*Δ/Δ mutants displaying the wild type hyphae, induced significantly different levels of IL-1β and macrophage lysis. Therefore, they concluded that hyphal development is not necessarily required for IL-1β production or macrophage lysis, and morphogenesis alone is insufficient to fully explain *Candida*-host interactions. Meanwhile, this study hypothesized that pyroptosis, a caspase-1-mediated programmed cell death process, might result in the production of IL-1 and the lysis of intracellular pathogens in macrophage [53]. Such hypothesis was further confirmed by a genome-wide analysis using the GRACE library. The mutants were submitted to hyphal-induction conditions to differentiate ones capable and incapable of filamentation and then co-cultured with macrophages to induce pyroptosis. Most hyphae-deficient mutants and certain mutants with normal hyphae were incompetent for induction of macrophage lysis, such as *tetO*-*RFT1*/*RFT1*Δ and *tetO*-*VPS35*/*VPS35*Δ mutants. Unexpectedly, some mutants, including *ALG1*, *ALG11,* and *orf19.6233* conditional expression strains, that are unable to form hyphae can still induce lysis. It was demonstrated that hyphal formation is not required for *C. albicans* to escape from host immune cells. Instead, macrophage pyroptosis is driven by fungal cell-wall remodeling and exposure of glycosylated proteins to macrophage phagosomes [21]. By using of the same mutant collection, it was found that the Hog1 MAPK signaling pathway is at the core of fungal cell-wall remodeling in response to macrophages. Meanwhile, it was also determined that the BCL10-MALT1 pathway is required to initiate inflammatory vesicles leading to host programmed cell death. Defects in pyroptosis activation would affect the recruitment of immune cells during infection. Such studies lay the groundwork for understanding *Candida*–host interactions [54].

*C. albicans* is the most common member of the intestinal flora, and its adaptation by colonization and invasion to the intestine is a complex process. Mutants in a library carrying unique sequence tags help to discriminate from each other in the mixed population. For example, after inoculating the mice by gavage with the mix of such mutants, researchers can identify the relative abundance of the strains collected from the stools. In a competitive gastrointestinal colonization experiment, the P*_TET_*-inducible expression library was inoculated in a mouse model. It was discovered that the transcription factor Crz2p directly regulates the expression of many mannoprotein genes and cell-wall-protein-encoding genes, leading to influencing cell wall function in *C. albicans*. High expression of *CRZ2* changed the phosphomannan abundance of cell wall and enhanced the adaptation to acidic environment and bile salt stimulation [25]. To elucidate the role of yeast and-hyphal form in gastrointestinal colonization, a *C. albicans* homozygous gene disruption library with barcodes for each mutant was gavaged in mice, and the survived colonies from feces were further sequenced. It was revealed that yeast-form mutant was prevalent in murine stomach and small intestines, while hyphal-form mutants were mainly located in ceca and large intestine. In addition, filamentations were found to inhibit commensalism. It was determined that the hyphal transcription factor Ume6p increases the expression of secreted protease *SAP6* and adhesion *HYR1*, therefore promoting virulence while inhibiting commensalism in gut [55].

The application of library makes it much easier to comprehend the interaction mechanism between fungal ecological dysregulation and the mucosal immune system, as well as its impact on the host in the setting of disease. These studies contribute to define new targets for reversing immunological dysfunction caused by fungal dysbiosis.

### 3.4. Antifungal Targets Identification

**Figure 2:**
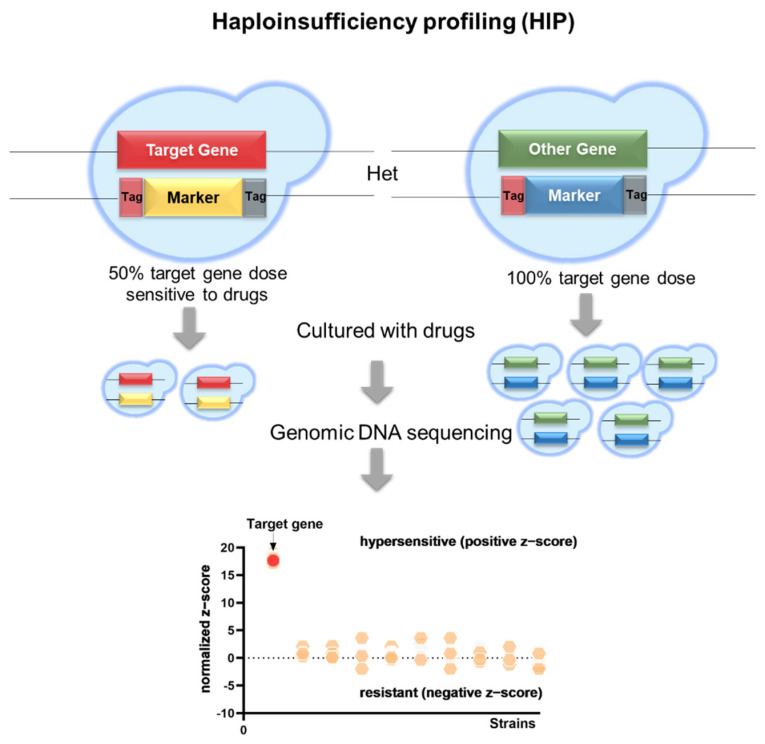
Schematic description of haploinsufficiency profiling (HIP). Heterozygous deletion mutants in a diploid were constructed by homologous recombination. Each strain was tagged with two unique DNA barcodes. The heterozygous mutant libraries were treated with antifungal drugs for dozens of doubling times. Then, genomic DNA were isolated, and the DNA barcodes were amplified by PCR. Finally, the barcodes were sequenced and normalized to the untreated pool. Het, heterozygous.

The incidence and mortality of invasive fungal infections have been continuously increased year by year, yet there are limited types of available antifungal agents and drug resistance is becoming a severe problem [56]. Therefore, it is necessary to identify new targets and develop novel antifungal drugs with high efficacy and a broad spectrum. Several mutant libraries are now available for screening compounds for antifungal activity and identifying antifungal targets, such as *S. cerevisiae* homozygous and heterozygous mutant libraries and the *C. albicans* GRACE library. Among them, haploinsufficiency profiles (HIP) based on the heterozygous mutant library is an effective means to identify novel antifungal compound targets. As shown in Figure 2, HIP was mainly dependent on the positively correlation between gene dose and drug sensitivity.

The *C. albicans* GRACE library was first used to screen the existing antifungal agents including fluconazole, tunicamycin, and 3-amino triazole. It was verified that strains with reduced expression of target genes, for example *ERG11*, *ALG3*, and *HIS3*, respectively, are hypersensitive to the corresponding agents. This preliminary study indicates the broad utility of this library for potential antifungal drug target screening [21]. Furthermore, a collection of 887 null mutants of non-essential genes derived from *C. albicans* GRACE library was used to study the mechanisms of action of the traditional antifungal drugs, including fluconazole, posaconazole, caspofungin, anidulafungin, amphotericin B, and the two new pyridine amides GPI-C107 and GPI-LY7. Similar sensitive or resistant mutants were identified to the same class of drugs, which demonstrated the reliability of such screening. This study also revealed that even though the apparent target of the medicine is the same among organisms, for example, in *S cerevisiae* or in *C. albicans*, drug sensitivity and resistance profiles might vary significantly, which emphasizes the necessity of construction of such collection in the pathogenic *C. albicans* [26]. More recently, Fu C et al., 2021, built a machine learning model for genome-wide prediction of genes’ importance in *C. albicans* based on the GRACE library. The model was also used to guide the generation of 866 additional GRACE mutants (called GRACEv2), further expanding the library and ultimately identifying 149 fungal-specific essential genes. Three essential genes, *KRP1*, *EMF1*, and *TIF33*, were defined to play roles in kinetochore function, mitochondrial integrity, and translation. In further chemical genetic analysis, after screening 9600 compounds, N-pyrimidinyl-β-thiophenylacrylamide (NP-BTA) was identified as being strongly active against *C. albicans* through inhibiting the essential glutamyl tRNA synthetase Gln4p [27]. Xu et al., 2007, constructed a library of 2868 heterozygous deletion *C. albicans* mutants and with 35 compounds tried to identify genes relevant to the probe compounds’ mechanism of action. This study demonstrated a way of identifying the mechanism of action of novel antifungal drugs using chemically induced haploid deficient spectra, verifying the technology’s potential usage in antifungal drug discovery [9]. Oh et al., 2010, constructed a library containing 3633 heterozygous transposon insertions and then identified 269 genes by haploinsufficiency profile analysis under four different nutritional conditions. In addition, Sec7p was discovered as the target of brefeldin A, while Tfp1p was found as a particular target of the synthetic chemical 0136-0228 by screening nearly 60 compounds. These findings underscore the importance of haploinsufficiency screening for genetic analysis and therapeutic target identification in *C. albicans* [18]. In order to discover the antifungal mechanism of chitosan in *C. albicans*, Shih PY et al., 2019, carried out mutation library screening [12,16,17,57,58] and found that chitosan could inhibit SAGA complex expression and lower the cell surface’s resistance to chitosan [23].

In addition to new antifungal target identification, large-scale genetic screening was also suitable for antifungal resistance studies. By creating a collection of 82 strains with high expression of transcription factors in *C. albicans* SC5314, Schillig was able to identify the regulatory factors of different virulence-related features. The transcription factor Mrr2p was proven to enhance azole resistance in *C. albicans* by upregulating the expression of *CDR1* [24]. A genome-wide screening with a collection of *Piggybac* transposon insertion mutation corresponding to 4791 nonessential genes identified that *fen1*Δ/Δ and *fen12*Δ/Δ mutants (encoding enzymes for the formation of very-long-chain fatty acids as sphingolipid precursors) were resistant to fluconazole and had greater levels of sphinolipid in cells. Further analysis revealed that *UPC2*, a gene involved in sphingolipid biosynthesis, was significantly overexpressed in both the wild-type and mutants, suggesting a novel mechanism of azole resistance [14]. Later, through the screening of the PB transposon insertion mutation library, Zeng G et al., 2021, discovered that *GPI7* deletion reduces the integrity of cell wall, thereby allowing compensatory chitin synthesis via the PKC-MAPK pathway but not *Rho1*-dependent, leading to caspofungin resistance [59].

Current studies showed that HIP analysis is the most effective means to identify novel antifungal drug targets. In 2020, the antifungal target identification of a novel and highly effective anti-*C. auris* compound turbinmicin still relied on the *S. cerevisiae* heterozygous and homozygous mutant libraries [60]. Therefore, constructing a pathogenic fungal library with more heterozygous and homozygous mutations is meaningful for the development of antifungal drugs.

## 4. Discussion

*C. albicans*, unlike *S. cerevisiae*, is frequently found in a diploid form in nature and does not have a traditional sexual cycle [42]. As a result, typical genetic manipulation approaches based on *S. cerevisiae* cannot be properly applied to the study of gene function in *C. albicans*. Until now, multiple *C. albicans* genetic mutant libraries have been established and screened for various phenotypes. Gene knockout libraries and transposon insertion libraries cover almost all non-essential genes, while conditional expression libraries also contain a large number of essential genes in *C. albicans*. These libraries have been widely used for large-scale genetic screening and genome-wide functional analysis, contributing to a profound understanding of morphogenesis, biofilm formation, host–pathogen interactions, and resistance to antifungal drugs. However, the knockout library relies on homologous recombination, which necessitates more than one screening marker and repeated transfections in one strain, consuming a lot of time and effort. The transposon gene library does not obtain homozygous deletion mutants that screening might miss numerous phenotypes. As for the newly developed CRISPR/Cas9 technology, off-targeting is an issue of concern. Therefore, it is also necessary to continuously improve and optimize the methods of gene library construction. In addition, how to properly preserve and share the data of *C. albicans* gene library and functional genome screening is an important issue. Researchers have been already working on developing a database for *C. albicans’* functional genomic screening. For example, Nicholas C et al., 2021, collated data from four mutant libraries of approximately 400 functional genomic screens in *C. albicans* to generate a database of genetic mutant strains [6,7,11,22], which provides a new resource for fungal genetics research. Unfortunately, there is currently no mutation library covering the whole genome for *Candida* [61]. If a genome-wide mutation database of *C. albicans* is built successfully, it will become a powerful tool for revealing the functional relationship between genes and improving our ability to explain gene networks on a broad scale, as well as accelerating the advancement of fungi research.

## Figures and Tables

**Table 1:** *C. albicans* mutant libraries for gene function studies.

Type of Mutation	Libraries and References	Methods	Parental Strains	Mutant Strains Generated	Genes Affected	Numbers of Alleles Affected	Application of Library
Gene disruption	Xu et al., 2007 [9]	gene knockout (*HIS1* and *LEU3* marker)	unreported	2868	69	Monoallelic	Identification of the mechanism of action of novel antifungal agents
	Homann et al., 2009 [6]	gene knockout (*HIS1*, *LEU2,* and *ARG4* marker)	SN152	365	143	Biallelic	Identification of regulators for drug resistance, morphological variation, and iron acquisition
	Noble et al., 2010 [7]	gene knockout (*HIS1* and LEU2 marker)	SN152	~3000	115	Biallelic	Morphogenetic switching and pathogenicity
	Shapiro et al., 2018 [8]	CRISPR–Cas9	GZY803, GZY892	250	250	Biallelic	Targeting antifungal efflux and biofilm adhesion factors
Transposon insertion	Davis et al., 2002 [12]	Tn7-UAU1	CAI4	353	3	Biallelic	Identification of pH-response regulators
	Uhl et al., 2003 [19]	Tn7-URA3	CAI4	18000	146	Monoallelic	Analysis of genes for filamentous growth
	Nobile and Mitchell et al., 2005 [16]	Tn7-UAU1	DAY185	83	2	Biallelic	Regulation of cell-surface genes and biofilm formation
	Norice et al., 2007 [17]	Tn7-UAU1	CAI4	25	21	Biallelic	Validation of essential genes for biofilm formation and virulence
	Oh et al., 2010 [18]	Tn5-UAU1	BWP17	3633	269	Monoallelic	Identification of gene annotation and drug target
	Blankenship et al., 2010 [11]	Tn7-UAU1	BWP17	108	80	Biallelic	Analysis of protein kinase signaling for cell wall regulation
	Epp E et al., 2010 [13]	Tn7-UAU1	SN95	4700	20	Biallelic	Identification of genes for morphological transformation
	Bharucha N et al., 2011 [10]	Tn7-URA3	*cbk1*Δ/*CBK1*	6528	441	Monoallelic	Analysis of the RAM Network during Morphogenesis
	Gao J. et al., (2018) [14]	PB-URA3	GZY892	4791	4791	Monoallelic	Identification of regulators of the white-to-opaque switching; identification of mutants resistant to caspofungin
	Mielich K et al., 2018 [15]	AS/Ds-NAT1	GZY896	1610	1195	Monoallelic	Analysis of *C. albicans* essential genes
Conditional expression	Roemer et al., 2003 [22]	GRACE	CaSS1	1152	567	Biallelic	Identification and prioritization of essential genes as antifungal drug targets
	Sahni et al., 2010 [23]	P_tet_ induced overexpression	P37005	107	107	Monoallelic	Analysis of the evolution of new signal transduction pathways
	Chauvel et al., 2012 [20]; Znaidi et al., 2018 [25]	P_tet_ and P_PCK1_ induced overexpression	BWP17	956	572	Monoallelic	Identification of regulators of morphogenesis and fitness; colonization of the mammalian GI tract; cell-wall function
	Schillig, R; Morschhäuser, J; et al., 2013 [24]	P_GAL4_ induced overexpression	SC5314	82	82	Monoallelic	Identification of regulators of invasive filamentous growth and mediators of fluconazole resistance
	O’Meara et al., 2015 [21]	GRACE	unreported	2356	974	Monoallelic	Global analysis of morphogenesis; macrophage pyroptosis
	Chen Y et al., 2018 [26]	GRACE	CaSS1	887	unreported	Biallelic	Screening GRACE collection of strains against classic antifungal drugs.
	Fu C et al., 2021 [27]	GRACE	CaLC6106	866	149	Monoallelic	Identification of fungal-specific essential genes and an antifungal compound that targets C. albicans glutaminyl-tRNA synthetase.

## Data Availability

The review did not report any data.
